# An early warning approach for the rapid identification of extreme weather disasters based on phased array dual polarization radar cooperative network data

**DOI:** 10.1371/journal.pone.0296044

**Published:** 2024-01-03

**Authors:** Miaoyuan Xiao, Lei Wang, Yuanchang Dong, Chenghong Zhang, Shunjiu Wang, Kangquan Yang, Kui Zhang

**Affiliations:** 1 Engineering Design & Research Institute of Sichuan University, Chengdu, China; 2 Chengdu Institute of Plateau Meteorology; CMA/Heavy Rain and Drought-Flood Disaster in Plateau and Basin Key Laboratory of Sichuan Province, Chengdu, China; 3 Sichuan Meteorological Disaster Prevention Technology Center, Chengdu, China; 4 Sichuan Climate Center, Chengdu, China; 5 Sichuan Meteorological Observatory, Chengdu, China; 6 Chengdu Meteorological Observatory, Chengdu, China; ICIMOD: International Centre for Integrated Mountain Development, NEPAL

## Abstract

In recent years, X-band phase array dual polarization weather radar technology has matured. The cooperative networking data from X-band phase array dual polarization weather radar have many advantages compared with traditional methods, namely, high spatial and temporal resolution (approximately 70 seconds in one scan, 30 m in radial distance resolution), wide coverages that can compensate for the observation blind spots, and data fusion technology that is used in the observation overlap area to ensure that the observed precipitation data have spatial continuity. Based on the above radar systems, this study proposes an improved hail and lightning weather disaster rapid identification and early warning algorithm. The improved thunderstorm identification, tracking, analysis, and nowcasting (TITAN) algorithm is used to quickly identify three-dimensional strong convective storm cells. Large sample observation experiment data are used to invert the localized hail index (*H*_*DR*_) to identify the hail position. The fuzzy logic method is used to comprehensively determine the probability of lightning occurrence. The comparative analysis experiment shows that, compared with the live observation data from the ground-based automatic station, the hail and lightning disaster weather warning algorithm developed by this study can increase warning times by approximately 7 minutes over the traditional algorithm, and its critical success index (CSI), false alarm ratio (FAR) and omission alarm ratio (OAR) scores are better than those of the traditional method. The average root mean square error (ARMSE) for identifying hail and lightning locations by this improved method is also significantly better than that of traditional methods. We show that our method can provide probabilistic predictions that improve hail and lightning identification, improve the precision of early warning and support operational utility at higher resolutions and with greater lead times that traditional methods struggle to achieve.

## Introduction

In recent years, small- and medium-scale extreme disaster weather events have occurred frequently worldwide. A large hailstorm occurred in Yarborough, Queensland, Australia, with the largest hailstone reaching 16 centimeters in diameter, breaking the country’s record for the largest hail [1]. On May 14, 2021, a tornado in Caidian District of Wuhan City killed 6 people and injured 218 [2]. On April 11,2022, a rare gale and hail weather event occurred in Anyue County, Sichuan Province, with a maximum wind force reaching up to level 13 (a severe hurricane), resulting in waterlogging, house collapse and serious damage to public facilities in some areas [3]. Only in Sichuan Province of China have there been nearly 40 mesoscale severe convective weather events in the past two years, especially extreme weather events such as hail, thunderstorms and tornadoes.

Extreme and severe weather disasters are characterized by small spatial scales, high strength, abruptness, rapid development and evolution, and considerable destructive power. X-band phased array dual-polarization weather radar (XPADPRDA), developed by China’s Narada Technology Company, has become the most unique and effective means for real-time monitoring and early warning of extreme disaster weather due to its fast electric scanning mode, array of antennas and high spatial and temporal resolution [4]. S-band weather radar (SRDA), which is currently widely used by China’s meteorological, civil aviation and hydrological departments, uses a mechanical scanning mode to complete a scan for approximately 6 minutes. However, XPADPRDA uses a phase array electrical scanning, which can quickly change the beam pitch angle of the antenna, and it takes only approximately 1 minute to complete a scan. In addition, due to the short wavelength, XPADPRDA greatly improves the spatial-temporal resolution of detection compared with SRDA [5, 6] and has the ability to detect polarization physical quantity data, so it has the ability to significantly improve the rapid identification and early warning accuracy of extreme disaster weather such as hail, thunderstorms and gales [7].

The National Oceanic and Atmospheric Administration (NOAA) and National Weather Service (NWS) in the United States specifically studied the data quality of the Weather Surveillance Radar-1988 Doppler (WSR-88D) dual-linear polarization radar, and its preliminary performance in monitoring and early warning was discussed at the IIPS conference held by the American Meteorological Society (AMS) in 2009. Islam et al. [8] noted that dual-polarization radar observations can accurately identify nearly ten kinds of precipitation particle phases when the fuzzy logic algorithm is applied to the data. Lei et al. [9] proposed a multiorder correlation estimation method to suppress the jitter interference of differential propagation phase return signals caused by X-band radar backscattering. Mahale et al. [10] used the fuzzy logic method to identify the three-body scattering echo characteristics of hail and remove false echo signals based on S-band dual-polarization radar data. Park et al. [11] noted that the (*K*_*dp*_) value of the differential propagation phase shift rate of dual-polarization radar is approximately linear with the attenuation rate of the reflectivity factor (*Z*_h_) and the differential reflectivity (*Z*_*dr*_). Compared with traditional single-polarization weather radar, dual-polarization radar can more accurately correct the *Z*_h_ and Z_*dr*_ attenuation values.

The US WSR-88D Severe Storm Laboratory has provided the SCIT storm identification and tracking algorithm for a long time [12]. On the other hand, the thunderstorm identification, tracking, analysis, and nowcasting (TITAN) system [13, 14] is a forecast system based on the radar observation system developed by the National Center for Atmospheric Research. The main algorithms of the TITAN system are widely used in America and other countries and regions in the world. Zhang et al. [15] used a non-single-threshold fuzzy logic method to identify hail. This method comprehensively considers the influence of multiple radar characteristic index parameters on the identification and early warning results but can only give a certain probability value and cannot give a certain result. Chen et al. [16] and Arora et al. [17] used a logistic regression model to invert the probability model of triggering events. On the basis of the above, Wang et al. [18] used the improved TITAN algorithm and logistic regression model to provide a rapid warning method for extreme weather disasters. Wang et al. [19] used S-band dual-polarization Doppler radar to detect tornadoes and hailstorms and provided a dual-polarization radar data identification and early warning hail and tornado algorithm.

On the other hand, due to the limitation of the mechanical scanning mode and the influence of the curvature of the Earth, the single SRDA currently makes the boundary layer convective weather system a blind spot for radar observation. Therefore, the construction of the XPADPRDA collaborative networking system with high spatial and temporal resolution can compensate for this deficiency. In 2003, the American Science Foundation established an engineering research center and jointly developed a distributed XPADPRDA cooperative networking system (CASA) for low-cost, low-power solid-state transmitters in conjunction with universities and IBM [20, 21]. After more than ten years of observation and testing, the CASA system has achieved remarkable results, especially for the fine detection of small- and medium-scale extreme disaster weather, which is obviously better than conventional single weather radar.

On the basis of the above research, this study carries out data quality control (QC) and collaborative network analysis on the observation data of extreme convective weather disasters over the past five years from three XPADPRDA systems deployed in Chengdu. The improved TITAN algorithm is used to quickly identify the three-dimensional strong storm cells from the networking system, and the hail index (*H*_*DR*_) and fuzzy logic method are used to identify the hail location and the probability of early warning lightning occurrence. Compared with other traditional works, the main improvements of this study are as follows: (1) The average warning time is increased by approximately 7 minutes; (2) the false alarm rate (FAR) and the false negative rate in the warning probability of the critical success index (CSI) are significantly reduced; and (3) the average root mean square error (ARMSE) identified and located is significantly reduced.

## Raw radar data processing

### Basic information

**Fig 1:**
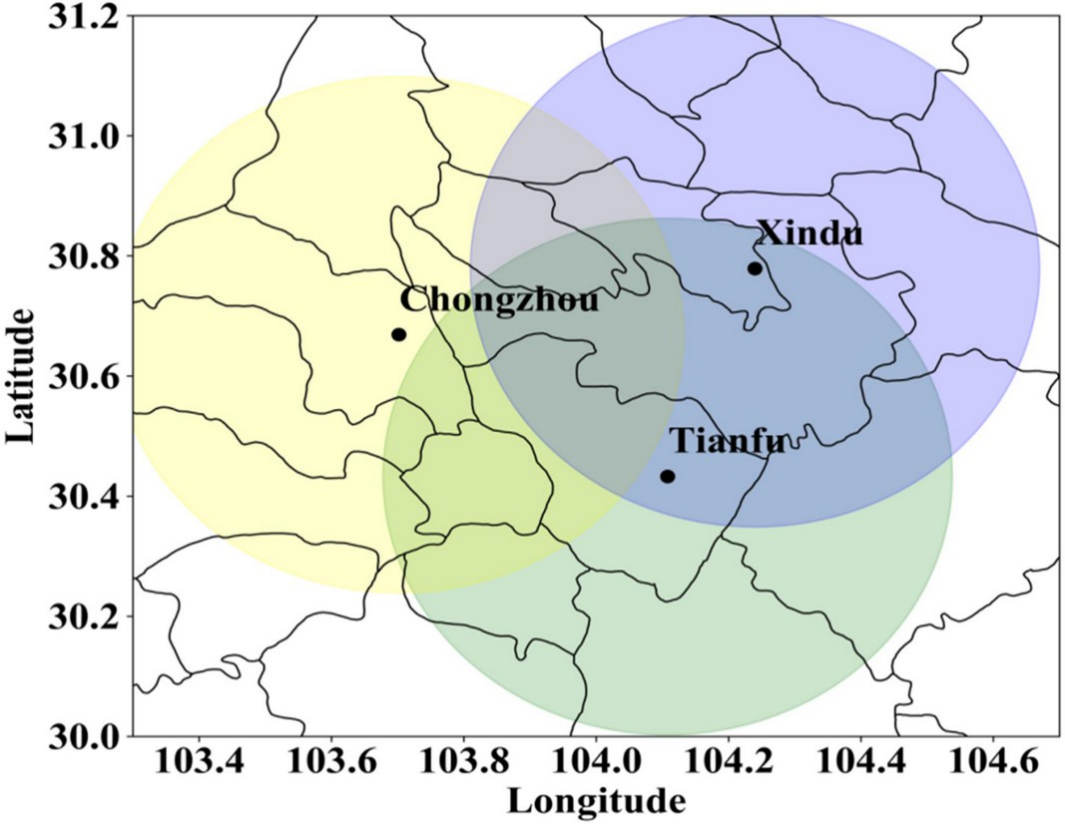
Distribution of the Chengdu XPADPRDA networking system (drawn with Python 3.6). Yellow circles represent the Chongzhou District radar, blue circles represent the Xindu District radar, and green circles represent the Tianfu New District radar.

Since 2017, the Chengdu XPADPRDA network has been built and put into observation experiments. The network system consists of three radars, which are located in Chongzhou District, Tianfu New District and Xindu District of Chengdu (Fig 1). The maximum detection radius of a single radar is 60 km, and the effective coverage area of the network reaches 12,000 square kilometers. The overlapping detection coverage area in the central urban area of Chengdu is 1728 square kilometers. The radar has a high spatial and temporal resolution. It can complete the three-dimensional radar detection data with a horizontal grid of 30 m × 30 m and with a vertical elevation interval of 1.5° in approximately 70 seconds. It can effectively improve the detection accuracy of extreme weather disasters and compensate for the detection blind spot of a single SRDA. Table 1 shows the observation performance parameters of the radar.

**Table 1:** Base parameters of X-band dual-polarization weather radar.

Parameter name	Parameter value	Unit	Definition
*λ*	3.2	*cm*	radar wavelength
*P*	50	w	peak power
*θ*_*h*_, *θ*_*v*_	1.22	°	horizontal beam width vertical beam width
	1.8		
PRF	1000	Hz	pulse repetition frequency
*τ*	80	*μs*	pulse width
*R*_max_	60	km	maximum sounding radius
*H*_max_	24	km	maximum sounding height
*V*_*r*_	-15~15	m/s	unambiguity velocity scope
*ρ*	30	m	radial distance resolution

### Radar data QC

Due to the complex terrain conditions in Chengdu and the short wavelength of XPADPRDA, there is interference from nonprecipitation echoes, substantial Φ_dp_ data jitter and *Z*_h_ and Z_*dr*_ attenuation problems in the original observation data. Therefore, it is necessary to properly perform XPADPRDA data QC. According to the principle of electromagnetic wave scattering [22], Φ_dp_ contains the forward scattering phase difference *φ*_*dp*_ and the backward scattering phase difference *δ*. The *δ* value increases with the increase in the equivalent diameter of precipitation particles and the detection distance, which results in the phase superposition effect of the *δ* value. This phenomenon interferes with the echo signal, producing the severe jitter of the Φ_dp_ value and further affecting the *K*_dp_ value. In addition, compared with SRDA, XPADPRDA has a shorter wavelength, so it will cause *Z*_h_ and *Z*_*dr*_ to greatly attenuate when observing heavy precipitation. Therefore, we must first control the quality of these dual-linear polarization physical quantity data. In this study, the mean square error calculated along the radial direction using continuous 5-point effective values is used to control the data quality (Eq 1):

$$\begin{array}{l} S=\sqrt{\frac{\sum_{\text{i}=1}^{5}{\left(\Phi_{dpi}-\bar{\Phi_{dp}}\right)}^{2}}{5}}\\ \left|\Phi_{dpi}-\Phi_{dpmid}\right|>S,\Phi_{dpi}=\Phi_{dpmid},\text{i}\in [1,5] \end{array}$$ (1)

**Fig 2:**
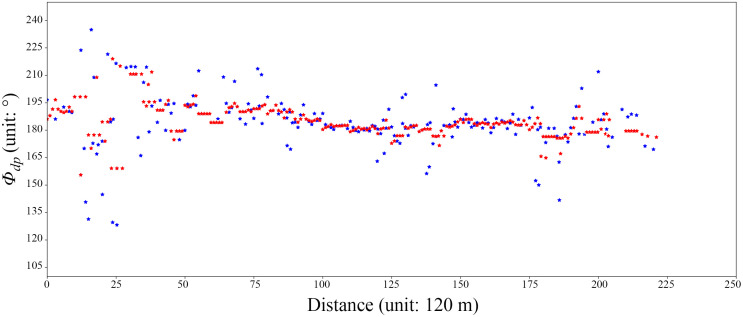
Quality control of Φ_dp_ at azimuth 125° on elevation 5° in Chongzhou district of 2020/03/31 13:21 (UTC). Blue stars are raw data, and red stars are QC data.

In Eq (1), S is the mean square error of the continuous 5-point effective values in the radial direction, and Φ_*dpmid*_ is the median value of the five points. By judging whether the absolute value of the difference between the 5-point Φ_dp_ value and the median is greater than the mean square difference, the phase difference jitter caused by the *δ* value is completed. Fig 2 shows the comparison of the effects of Φ_dp_ data before and after QC in strong convective precipitation observed by XPADPRDA in the Chongzhou District of Chengdu. After QC, the phase difference noise and jitter interference caused by the *δ* value are noticeably suppressed. On the other hand, the specific differential phase (*K*_dp_) parameter is introduced to better reflect the change in the phase difference of precipitation particles per unit distance. *K*_*dp*_ is defined as follows:

$$K_{\text{dp}}=\frac{\left(\Phi_{{\text{dp}}_{i+1}}-\Phi_{dp_{i}}\right)}{2*\left(r_{i+\text{1}}-r_{i}\right)}$$ (2)

Eq (2) shows that what is actually expressed by *K*_dp_ is the change rate of Φ_dp_ per unit distance. Therefore, QC over Φ_dp_ is helpful for the acquisition of more precise *K*_dp_ data.

When XPADPRDA detects heavy precipitation particles, especially hail, *Z*_h_ and Z_*dr*_ will produce substantial attenuation. According to many studies [23], the decay rates *A*_h_ of *Z*_h_ and *Z*_*dr*_ are linear with *K*_dp_. Therefore, we can use the abovementioned *K*_dp_ value after QC to correct the attenuation of *Z*_h_ and Z_*dr*_.

**Fig 3:**
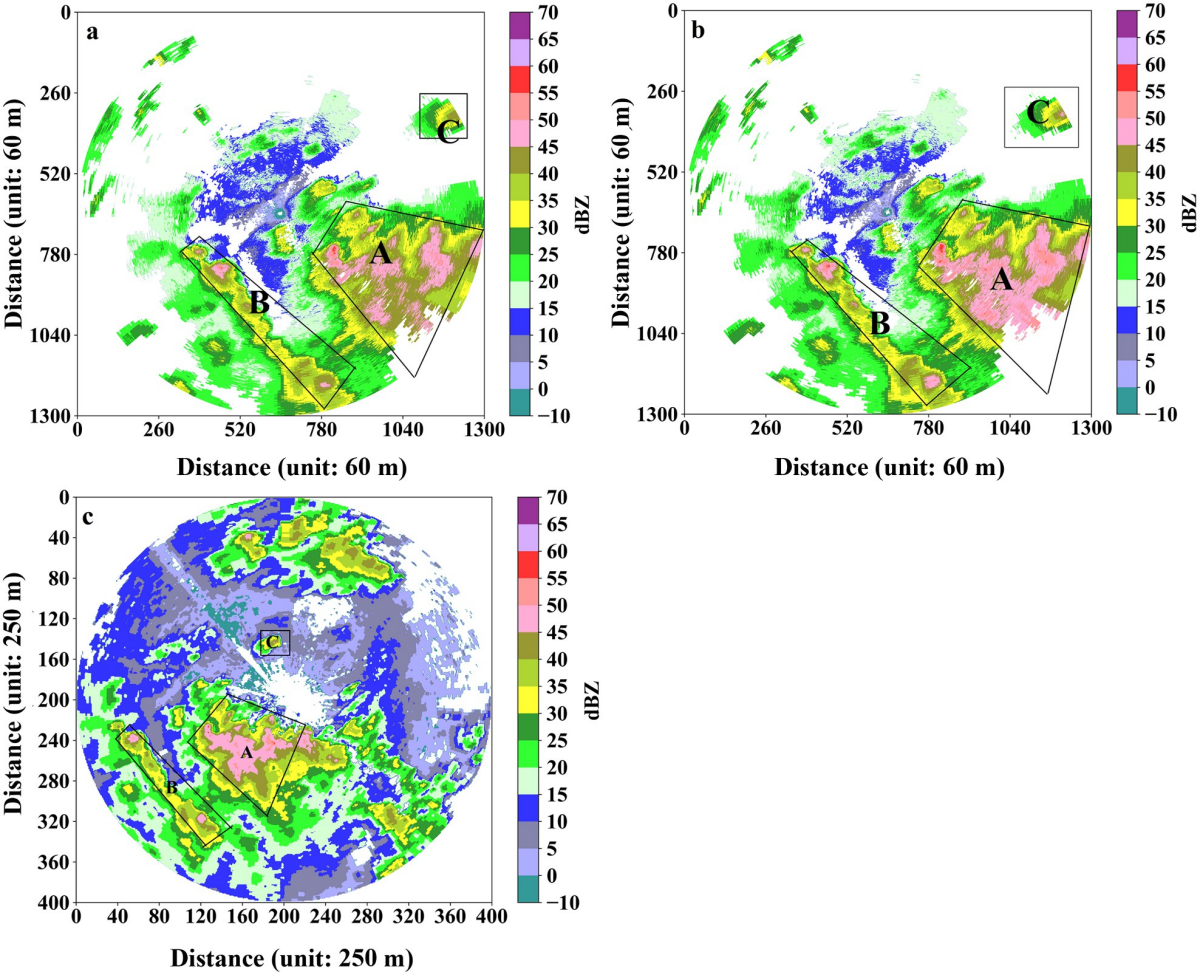
The contrast between before and after *Z*_h_ attenuation on PPI mode value attenuation at the minimum elevation (drawn with Python 3.6). (a) Raw *Z*_h_ data at the minimum elevation from June 16, 2020 17:23 (UTC). (b) Attenuation *Z*_h_ data at the minimum elevation from June 16, 2020 17:23 (UTC). (c) *Z*_h_ data at the minimum elevation from June 16, 2020 17:21 (UTC) with S-band radar of Chengdu.

According to the *K*_dp_-*A*_h_ linear relationship for the XPADPRDA systems in the Chengdu area [23], the *Z*_h_ attenuation value of the XPADPRDA observations in the Chongzhou area is corrected. Fig 3 shows the PPI distribution maps of the *Z*_h_ value at an elevation of 1.5° by two radars almost simultaneously. Fig 3a is the raw *Z*_h_ value observed by the XPADPRDA in the Chongzhou District, while Fig 3b is the corresponding *Z*_h_ value after attenuation correction. Fig 3c shows the *Z*_h_ value in Chengdu observed by the SRDA radar. The locations of the strong echo areas (marked A, B and C) within the three black wireframes are identical across the panel figures. With the *Z*_h_ value observed by the SRDA as the standard (Fig 3c), the *Z*_h_ value of the strong echo areas after attenuation correction (Fig 3b) is noticeably increased, which is closer to the value in Fig 3c than that in Fig 3a.

When the XPADPRDA system is observed, the incident angle of the vertical polarization beam changes with the elevation angle, so that the Z_*dr*_ value decreases exponentially with the elevation angle of the antenna. The larger the actual Z_*dr*_ value is, the faster the decrease. Therefore, the attenuation correction of the Z_*dr*_ value is more complicated. We uniformly correct the Z_*dr*_ values measured at different elevation angles to the Z_*dr*_ value at 0° elevation (Eq 3):

$$Z_{\text{dr}}(0)=\frac{{\text{cos}}^{4}\alpha *Z_{dr}(\alpha )}{{\left(1-\sqrt{Z_{dr}(\alpha )}{\text{sin}}^{2}\;\alpha \right)}^{2}}$$ (3)

In Eq (3), *Z*_*dr*_(*α*) represents the Z_*dr*_ value measured at different elevation angles, and *Z*_*dr*_(0) represents the Z_*dr*_ value uniformly corrected to a 0° elevation angle. After completing the elevation correction of Z_*dr*_, the attenuation correction is further performed, which is defined as follows:

$$\left\{\begin{array}{l} A_{\text{dr}}(r)=0.04916K_{\text{dp}}(r)\\ Z^{*}{}_{dr}(r)=Z_{dr}(r)+2\int_{0}^{\text{r}}{\text{A}}_{\text{dr}}(r)dr \end{array}\right.$$ (4)

**Fig 4:**
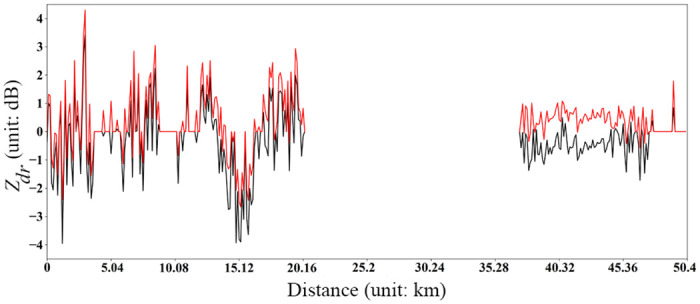
*Z*_dr_ attenuation correction of a convective cloud precipitation at azimuth 24° on an elevation of 5° in Chongzhou district of 2021/07/14 10:33 (UTC). The black line indicates the raw data and the red line is the data after quality control.

In Eq (4), *A*_dr_(*r*) represents the Z_*dr*_ attenuation rate at radial distance *r*, $Z_{dr}^{*}(r)$ is the actual differential reflectivity value after attenuation correction, and 0.04916 is the coefficient of the linear relationship of XPADPRDA *A*_dr_~*K*_dp_ in the Chengdu area that has been both observed and derived in the relevant literature [23]. Fig 4 shows the *Z*_dr_ data within a radial distance of 0–50 km obtained by the XPADPRDA at an azimuth of 24° for a convective cloud precipitation process that occurred in the Chongzhou District at 10:33 (UTC) on July 14th, 2021. In this figure, the black line indicates the raw *Z*_dr_ data, while the red line represents the corresponding *Z*_dr_ data after QC. It is also noteworthy that there is no line within the radial distance range from 21 km to 36 km as a consequence of no precipitation within this range. After QC, the *Z*_dr_ data increase markedly. Additionally, for a location relatively far away from the radar station in terms of radial distance (approximately 37–48 km), the corrected *Z*_dr_ value displays an exponential increase with the increase in the radial distance, which approaches the actual value.

**Fig 5:**
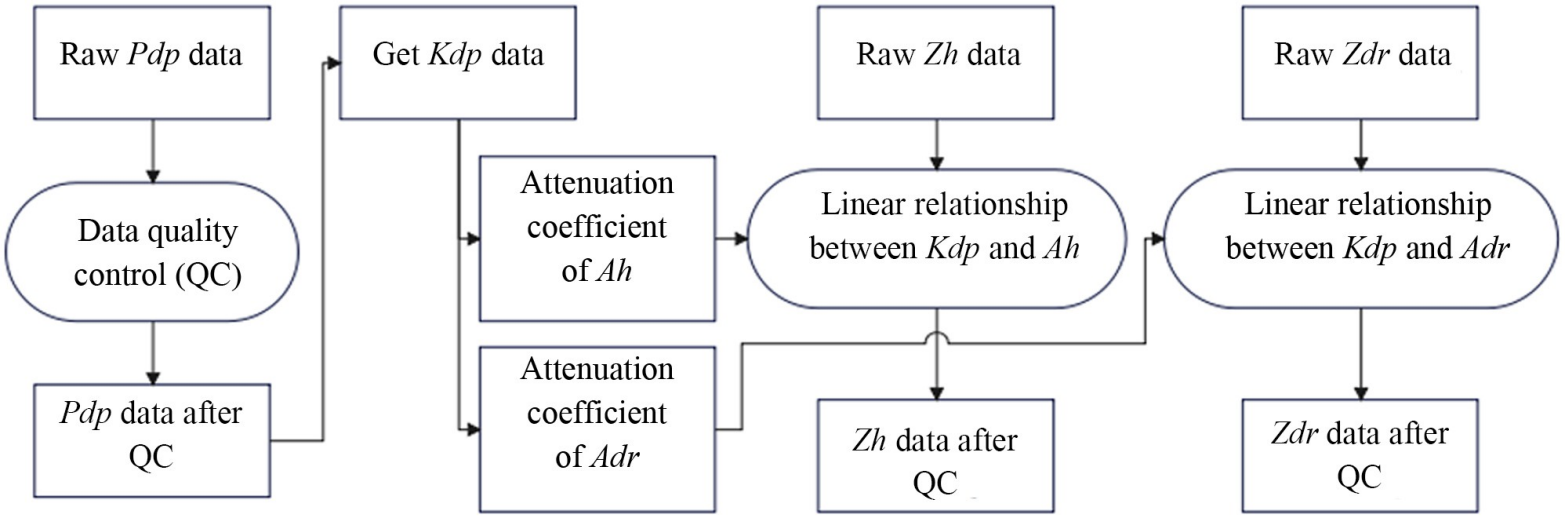
Relationships among the key polarization physical quantities (Φ_dp_, K_dp_, Z_dr_, Z_h_).

Fig 5 shows the relationships among the abovementioned key polarization physical quantities (Φ_dp_, K_dp_, Z_dr_, Z_h_). According to this figure, QC over the noise and jitter should be performed first for the raw Φ_dp_ data. Then, the K_dp_ value should be calculated according to definition, followed by the acquisition of the Z_h_, Z_dr_ value after attenuation correction based on the relationship between K_dp_ and the attenuation coefficients A_h_, A_dr_.

### XPADPRDA collaborative network

Using multiple XPADPRDA stations to construct a cooperative adaptive radar network system can effectively solve the problem that the detection ability of a single radar decreases with increasing detection distance. On the other hand, it can also solve the problems of blind spots in the boundary layer of convective precipitation events and ground object occlusion in complex mountainous terrain. In this study, three XPADPRDA radars in Chengdu are used for cooperative networking according to the parameters in Table 2.

**Table 2:** Parameters of the X-band full-polarization weather radar collaborative network.

Parameter name	Parameter value	Unit	Definition
Max_Range	138*138	km	The maximum distance span of radar network
Max_Grid	2300		The maximum number of grid points of radar network
Reso	0.01046*0.06	°/60 m	Longitudinal resolution Latitudinal resolution
	0.009*0.06		
cz_center	103.702, 30.6688	°	Chongzhou radar location
xd_center	104.24,30.7787	°	Xindu radar location
tf_center	104.108,30.4326	°	Tianfu radar location
net_center	104.017, 30.627	°	Network center location
radius	39	km	The maximum radius of actual detection of single radar
R_max	650		The effective detection radius grid points of single radar

Using the four-point inverse distance weight interpolation method [24], the radar data in the polar coordinate system are transformed into the Cartesian coordinate system (after the steps described in the radar data QC section have been applied). Eq 5 is used to project three XPADPRDA coordinates onto the actual coordinates of the cooperative network.


$$\left\{\begin{array}{l} x=(x^{\prime }-R_{\text{max}}^{\prime })+x_{mid}+\left[(long_{mid}^{\prime }-long_{mid})\times \frac{\pi R_{\text{e}arth}}{180}\text{cos}lat_{mid}^{\prime }÷\text{Re}\;so\right],0\leq \text{x}<2300\\ y=(y^{\prime }-R_{\text{max}}^{\prime })+y_{mid}-\left[(lat_{mid}^{\prime }-lat_{mid})\times \frac{\pi R_{\text{e}arth}}{180}÷\text{Re}\;so\right],0\leq \text{y}<2300 \end{array}\right.$$ (5)

In Eq 5, (x′, *y*′) represents the original coordinate positions of three single-station radar data points, and (x, *y*) is the actual coordinate position projected onto the network. The net _ center parameter in Table 2 represents the latitude and longitude of the center point of the three radar networking areas, which is represented by (long_*mid*_, *lat*_*mid*_) in Eq 5. In Eq 5, (long_*mid*_, *lat*_*mid*_) represents the Cartesian coordinates of the network center point, which is actually half of the value of the parameter Max_Grid in Table 2. In addition, R_max in Table 2 represents the effective detection radius range of a single radar, which is represented by (${\text{R}}_{\text{max}}^{\prime }$) in Eq 5. The Reso parameter in Table 2 also represents the range resolution that the networked radars can reach (60 m), which is represented by (Reso) in Eq 5. On the other hand, Eq 5 uses (${\text{long}}_{mid}^{\prime },lat_{mid}^{\prime }$) to represent the latitude and longitude of each single-station radar center point, namely, cz_center, xd_center and tf_center in Table 2. *R*_*earth*_ is a constant and represents the radius of the earth.

For the multiradar detection overlap area of the network, we use the exponential distance weight method [25] for data fusion processing (Eq 6). For a radar detection blind area (invalid value), the non-invalid value of other radars at the same position can be used to fill it. In this way, we can ensure the continuity of the original precipitation spatial structure as much as possible.


$$\begin{array}{l} D1=\sqrt{\left(x^{\prime }-x_{mid\;1}\right)*\left(y^{\prime }-y_{mid\;1}\right)}\\ D2=\sqrt{\left(x^{\prime }-x_{mid\;2}\right)*\left(y^{\prime }-y_{mid\;2}\right)}\\ w_{n}=e^{-\frac{Dn^{2}}{10000}},\text{n}=1,2\\ Zrel=\frac{w_{1}*Z_{1}+w_{2}*Z_{2}}{w_{1}+w_{2}} \end{array}$$ (6)

**Fig 6:**
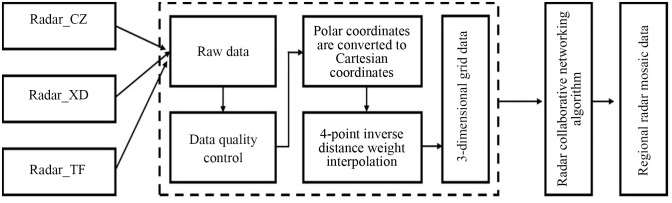
Three XPADPRAD of the Chengdu collaborative network mosaic process.

In Eq 6, D1, D2 is the distance between the radar data in the overlapping area and the respective radar station, w_n_ is the exponential weight coefficient, Z1, *Z*_2_ is the reflectivity factor value of the radar observation in the overlapping area, and Z_rel_ is the reflectivity factor value of the network after data fusion. Z_dr_ and correlation coefficient (*ρ*_hv_) values are treated the same as the Z_h_ value. Fig 6 shows the three XPADPRDA collaborative networking puzzle processes in Chengdu.

**Fig 7:**
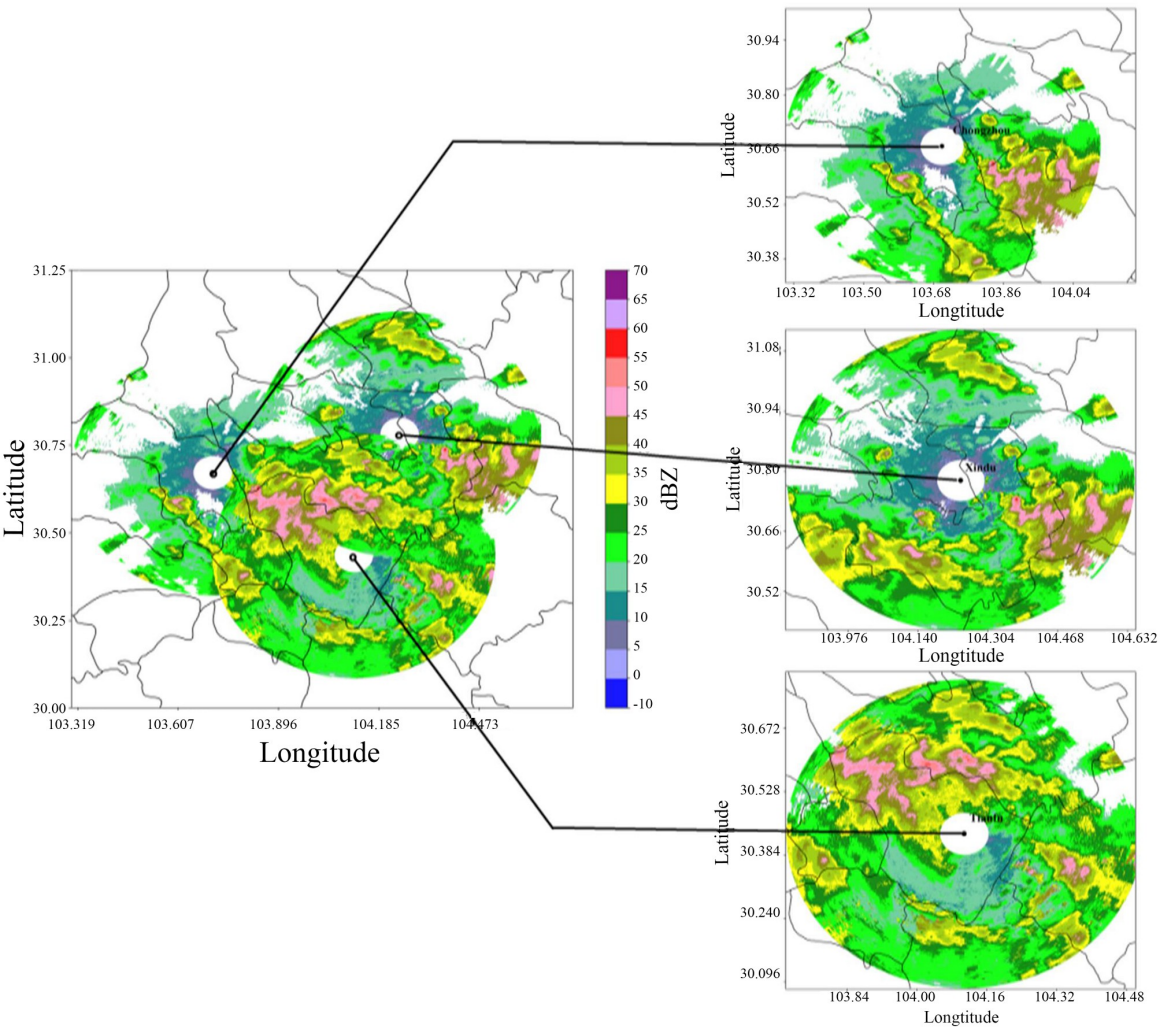
A typical severe convective precipitation process from the collaborative network mosaic from three XPADPRAD systems in Chengdu (drawn with Python 3.6).

Fig 7 shows the mosaic effect of three XPADPRDA cooperative networks at a height of 2 km during a typically strong convective precipitation process in the Chengdu area. Fig 7 shows that the coverage area of the reflectivity factor data after networking is significantly increased, and the whole strong convective precipitation echo area is more complete. Therefore, the voids near the radar station formed by the conical volume scanning interpolation of the three single-station radars are partially filled. The severe ground object occlusion near the radar station in Xindu District is filled by Chongzhou radar data. In the area of overlapping network data, due to the data fusion processing method, the area where the original echo is obviously stronger is suppressed, and the echo area where the original attenuation is stronger is corrected and enhanced. The whole convective precipitation system is more continuous in space than would otherwise be indicated by a single radar system.

### Hail and lightning identification and warning method

In severe convective precipitation systems, extreme disastrous weather events (such as hail, lightning and mesocyclones) are sometimes present. Some of the previous studies [26, 27] were based on single reflectivity data detected by monopolar weather radar. The basic step is to first identify the three-dimensional strong storm cell and then calculate the radar secondary products, such as the 30 dBZ and 40 dBZ strong echo height, echo top height (ET), vertically integrated liquid water content density (VIL), maximum reflectivity factor and three-dimensional storm centroid height. Second, a fixed threshold is set to comprehensively judge and identify the probability of hail and lightning; finally, the hail and lightning warning levels are given according to the probability value. However, dual-linear polarization radar contains more abundant polarization physical quantity detection data, which contain more basic information on the size, shape, concentration and phase of the hydrometeor. Therefore, this study introduces the *H*_*DR*_-index and uses the fuzzy logic method to jointly identify hail locations and lightning occurrence probabilities.

Tables 3 and 4 show the 53 sets of Z_h_, *Z*_dr_, *ρ*_*hv*_ regarding extreme severe convective weather disasters with hail (27 samples) and lightning (26 samples) observed by three XPADPRDA systems in Chengdu from June to September 2019 to 2021, respectively. Additionally, the ground-based automatic weather station and lightning locator are used to verify whether hail and lightning actually occur at the same time and position. Based on the observed experimental sample data in these tables, we collect approximately 15,500 sets of XPADPR-observed datasets for hail and lightning events in the Chengdu region between 2017 and 2022 to fit and invert the parameters of the hail *H*_*DR*_ index model and the lightning fuzzy logic membership function model.

**Table 3:** The observation experimental outcomes of three XPADPRDA systems for 27 hail events in the Chendu region between June and September from 2019 to 2021.

Sample number	Z_h_(dBZ)	*Z*_*dr*_(dB)	*ρ*_*hv*_	Is hail or not?
1	46.4	0.51	/	yes
2	34.5	-1.2	/	no
3	41.4	2.4	/	no
4	48.3	0.3	/	yes
5	41.4	1.5	/	no
6	55.8	1.17	/	yes
7	58.3	1.43	/	yes
8	45.6	1.36	/	no
9	61.5	0.87	/	yes
10	40.5	0.44	/	no
11	48.9	-0.16	/	yes
12	46.3	3.1	/	no
13	43.7	-0.66	/	yes
14	48.5	1.83	/	no
15	32.4	-0.8	/	no
16	34.7	-1.2	/	no
17	49.2	0.74	/	yes
18	53.4	0.66	/	yes
19	64.7	1.65	/	yes
20	42.6	1.47	/	no
21	44.5	1.12	/	no
22	51.3	0.94	/	yes
23	44.6	-0.45	/	yes
24	45.8	-1.24	/	yes
25	36.5	0.53	/	no
26	39.3	1.04	/	no
27	60.3	1.92	/	yes

**Table 4:** The observation experimental outcomes of three XPADPRDA systems for 26 lightnings in the Chendu region between June and September from 2019 to 2021.

Sample number	Z_h_(dBZ)	*Z*_*dr*_(dB)	*ρ*_*hv*_	Is lightning or not?
1	36.5	-2.2	0.97	yes
2	31	0.4	0.91	no
3	41	1.2	0.83	no
4	28.4	0.08	0.99	yes
5	33.5	-1.04	0.98	yes
6	38.2	-5.1	0.79	no
7	36.5	1.38	0.94	no
8	26.7	0.26	0.96	yes
9	31.4	-1.78	0.93	yes
10	43.5	2.65	0.95	no
11	29.6	-1.87	0.96	yes
12	31.5	-2.1	0.93	no
13	32.4	-0.53	0.99	yes
14	41.4	1.87	0.97	yes
15	38.3	0.45	0.96	no
16	36.5	0.65	0.97	yes
17	42.7	-0.63	0.92	yes
18	34.5	1.24	0.95	yes
19	45.8	1.92	0.95	no
20	27.5	0.32	0.98	yes
21	26.4	0.44	0.85	no
22	28.9	1.06	0.89	yes
23	39.7	1.41	0.98	no
24	47.3	2.32	0.97	no
25	36.4	0.72	0.93	yes
26	51.6	2.07	0.96	no

Based on observation data from the XPADPRDA cooperative network, the circular recursive region growing method is used to quickly identify the two-dimensional storm surface of radar echoes at different heights. According to the correlation of the centroid distance of the two-dimensional storm surfaces at adjacent heights, the three-dimensional convective storm cell is further constructed. Table 5 shows the identification threshold parameters of severe convective storm cells in Chengdu. Only when all the first identified three-dimensional storm cells meet the threshold conditions in Table 5 can they be used as the actual strong storm monomer.

**Table 5:** Three-dimensional convective storm identification parameters by XPADPRDA in the Chengdu area.

Parameter name	Parameter threshold	Unit	Remark
Maximum grid numbers of east–west direction	2300		Number of distance grid, needs to be calculated according to the maximum measurement range and radial distance resolution of the network.
Maximum grid numbers of south–north direction	2300		Number of distance grid, needs to be calculated according to the maximum measurement range and radial distance resolution of the network
Number of elevation layers	20		The total number of elevation layers of the XPADPRDA radar
Grid resolution	60	m	Minimum radial distance resolution
Maximum detection range	138	km	The maximum span distance from east to west or south to north
Maximum sounding height	10	km	The vertical height relative to the station altitude
Vertical height resolution	1	km	The interval of each height layer
Minimum reflectivity factor	35	dBZ	The lowest reflectivity used to determine whether a strong convection echo is true or not
Minimum two-dimensional storm area	8	km^2^	The smallest two-dimensional storm area
Centroid correlation distance	5	km	The maximum value of centroid distance of two-dimensional storm surface on adjacent heigh
Storm vertical extension thickness	3	km	The minimum extended thickness of three-dimensional convective storm cell
Minimum storm cell volume	60	km^3^	The smallest three-dimensional storm cell volume

**Fig 8:**
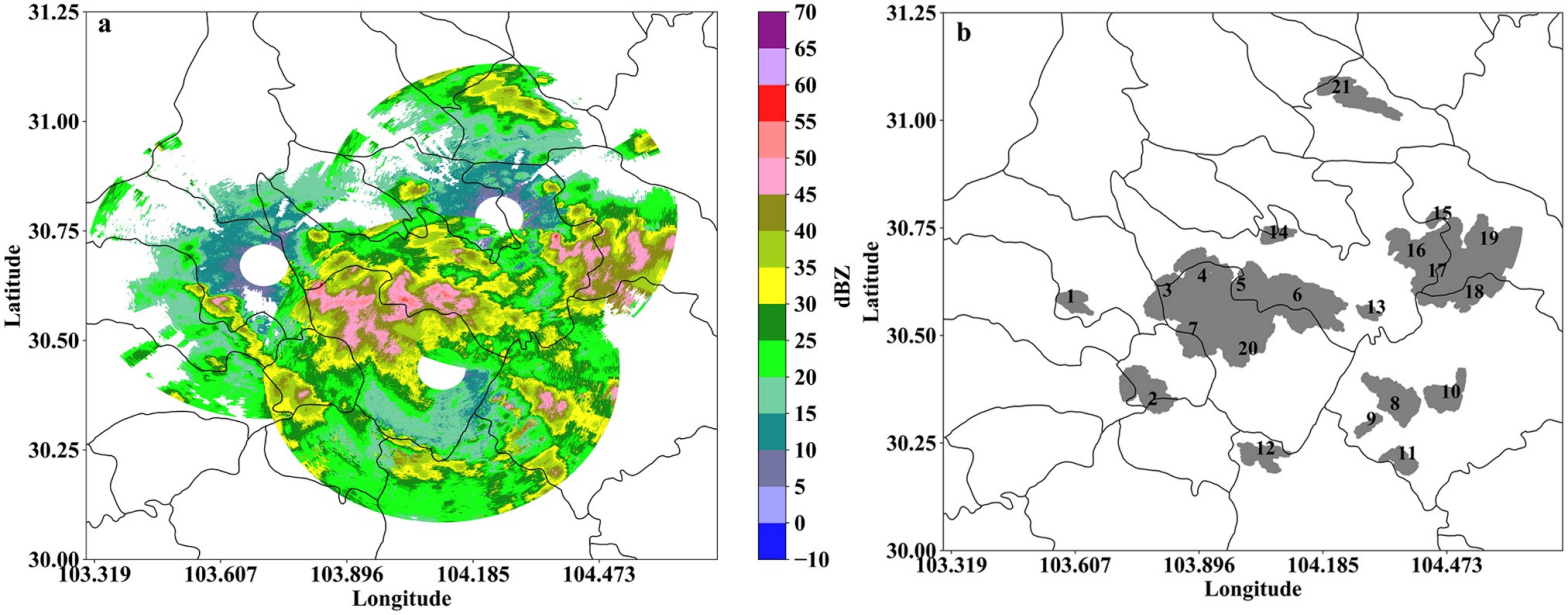
An extreme hail and lightning weather event occurred in Chengdu at 17:23 (UTC) on June 16, 2020. (a) Z_h_ observation data from the XPADPRDA collaborative network. (b) Identification results of three-dimensional strong convective storm cells based on network observation data in Fig 8a (gray blocks, 21 in total).

Fig 8 shows an extreme hail and lightning weather event that occurred in Chengdu at 17:23 (UTC) on June 16, 2020. Fig 8a shows the Z_h_ observation data from the XPADPRDA collaborative network. The improved TITAN algorithm presented in this study is used to quickly identify 21 three-dimensional strong convective storm cells and show the projection area of these storm cells at a height of 2 km (gray area in Fig 8b). Fig 8 shows that the echo intensity and area of these storm cells are different; some are isolated, and some are connected. Based on these results, we will further identify the location of hail and the probability of early warning lightning.

Based on the above improved TITAN algorithm to identify the three-dimensional strong storm monitoring results, this study will use the double linear polarization observation physical quantity and use the H_DR_ index [28] and fuzzy logic method to identify hail and lightning quickly and effectively. The H_DR_ index is defined as follows (Eq 7):

$$\begin{array}{l} \left\{\begin{array}{l} 35,\;Z_{dr}<0\\ 35+\alpha Z_{dr},0\leq Z_{dr}<1.6\\ 55,\;Z_{dr}\geq 1.6 \end{array}\right.\\ H_{DR}=Z_{h}-f\left(Z_{dr}\right) \end{array}$$ (7)

The α value in Eq 7 is variable according to different seasons and terrain conditions. In this study, according to the experimental observation data in the Chengdu area in Tables 3 and 4, α = 11.83 is obtained via inversion and fitting. The H_DR_ value of each point of each three-dimensional storm cell at a height of 2 km is calculated. When H_DR_ >> 0, there is hail at this point.

In addition, it is generally believed that charge separation in thunderstorm clouds requires a continuous upward flow. By judging whether the radar strong echo reaches a higher elevation, it can be inferred that there is such a strong and continuous upward flow at this position. Therefore, the volume proportion of strong radar echoes at a higher elevation has certain indicative significance for lightning identification and early warning. On the other hand, Carey et al. [29] also noted that polarization physical quantities such as Z_dr_ and ρ_hv_ are sensitive to the probability of lightning occurrence. In summary, this study combines the experimental observation data in Tables 3 and 4 and uses the fuzzy logic method to obtain the probability model of lightning identification and early warning in Chengdu. We calculate and obtain the Z_h_, Z_dr_, and ρ_hv_ values of each point of each three-dimensional storm cell at a height of 2 km and use the following functions to calculate the lightning probability value Pro for these three variables:

$$\begin{array}{l} \left\{\begin{array}{l} \frac{V_{\geq 35dBZ}^{3km}}{V}\geq 0.1,\;\text{Pr}\;o1=1\\ 0.05\leq \frac{V_{\geq 35dBZ}^{3km}}{V}<0.1,\;\text{Pr}\;o1=0.5\\ \frac{V_{\geq 35dBZ}^{3km}}{V}<0.05,\;\text{Pr}\;o1=0 \end{array}\right.\\ \left\{\begin{array}{l} Z_{dr}\leq -4,\;\text{Pr}\;o2=0\\ -4<Z_{dr}\leq -2,\;\text{Pr}\;o2=0.5*Z_{dr}+2\\ -2<Z_{dr}\leq -0.5,\;\text{Pr}\;o2=1\\ -0.5<Z_{dr}\leq 0.5,\;\text{Pr}\;o2=-Z_{dr}+0.5\\ Z_{dr}>0.5,\text{Pr}\;o2=0 \end{array}\right.\\ \left\{\begin{array}{l} \rho_{hv}\geq 0.96,\;\text{Pr}\;o3=1\\ 0.9\leq \rho_{hv}<0.96,\;\text{Pr}\;o3=16.67*\rho_{hv}-15.003\\ \rho_{hv}<0.9,\;\text{Pr}\;o3=0 \end{array}\right.\\ \text{Pro}=0.5*\text{Pro}1+0.25*(\text{Pro}2+\text{Pro}3) \end{array}$$ (8)

**Fig 9:**
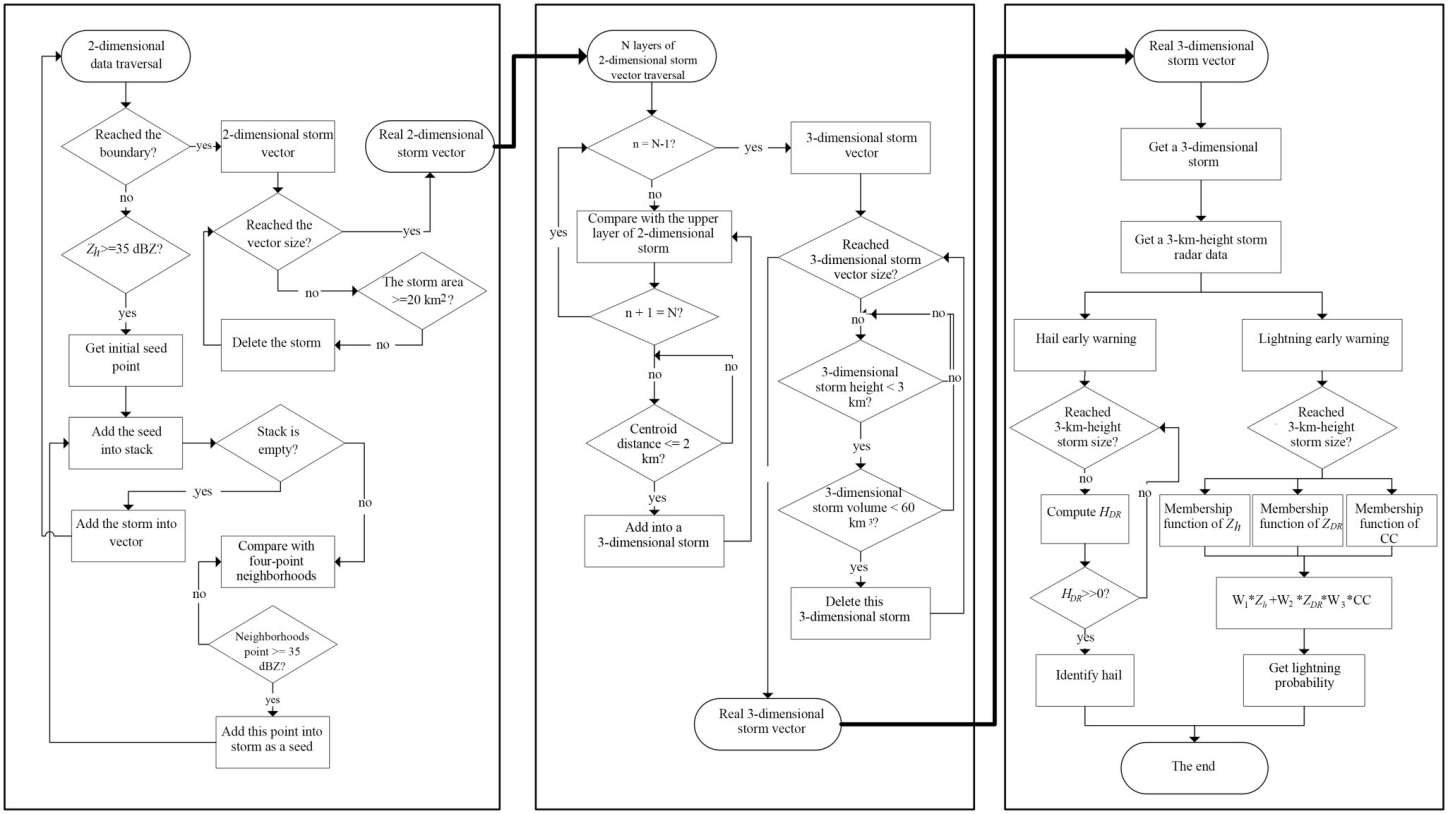
The total process of identifying hail and lightning extreme weather disasters and the early warning algorithm.

Fig 9 shows the process of hail and lightning disaster weather identification and the early warning algorithm. Fig 9 shows that the left block diagram uses the cyclic recursive region growth method to quickly identify the two-dimensional storm surface at different heights. The middle block diagram uses the centroid distance correlation method to construct the initial three-dimensional strong storm cell for the two-dimensional storm surface identified by the left block diagram and filter the final three-dimensional strong convective storm cell according to the threshold parameters in Table 5. The right block diagram is based on the actual three-dimensional storm cell results obtained from the intermediate block diagram, and the H_DR_ index and fuzzy logic method are used to identify the hail location and the probability of early warning lightning.

## Results and discussion

### Weather radar experimental observation data

A large-scale hail and lightning weather event occurred at 17:23 (UTC) on June 16, 2020, in Chengdu, Sichuan Province. To compare the experimental analysis with the observation data from the three XPADPRAD monitoring systems in the Chengdu collaborative network, we selected the SRDA observation data from the Chengdu operation that were acquired at the same time and location. The radar was built in 2004 and has been in operation since. The performance parameters of the radar during this weather observation are shown in Table 6.

**Table 6:** Performance parameters of S-band doppler weather radar.

Parameter name	Parameter value	Unit	Definition
*λ*	10	*cm*	radar wavelength
*P*	>10	kw	pulse power
*θ*_*h*_, *θ*_*v*_	0.99	°	horizontal beam width vertical beam width
	0.99		
PRF	1500	Hz	pulse repetition frequency
*τ*	1.5	*μs*	pulse width
*R*_real_	150	km	valid sounding radius
*H*_max_	20	km	maximum sounding height
*V*_*r*_	-27~27	m/s	unambiguity velocity scope
*ρ*	250	m	radial distance resolution
Lon	104.0159	°	Radar station longitude
Lat	30.6634	°	Radar station latitude

Table 6 shows that the SRDA wavelength is longer, and the effective observation range and velocity monitoring range are larger than those of the single XPADPRAD radar. Due to the lack of phased array dual-polarized antenna transceiver technology, there are no polarization detection data. Its radial detection range resolution is also significantly lower than that of the XPADPRAD radar. In addition, the radar station is located at 104.0159° E and 30.6634° N, which is very close to the central point of the cooperative networking in Table 2 (104.017° E and 30.627° N). In summary, this radar can better match the XPADPRAD network detection data in time and space, which is conducive to the comparative analysis of the experimental results of different identification and early warning algorithms.

### Ground-based automatic station live observation data

**Fig 10:**
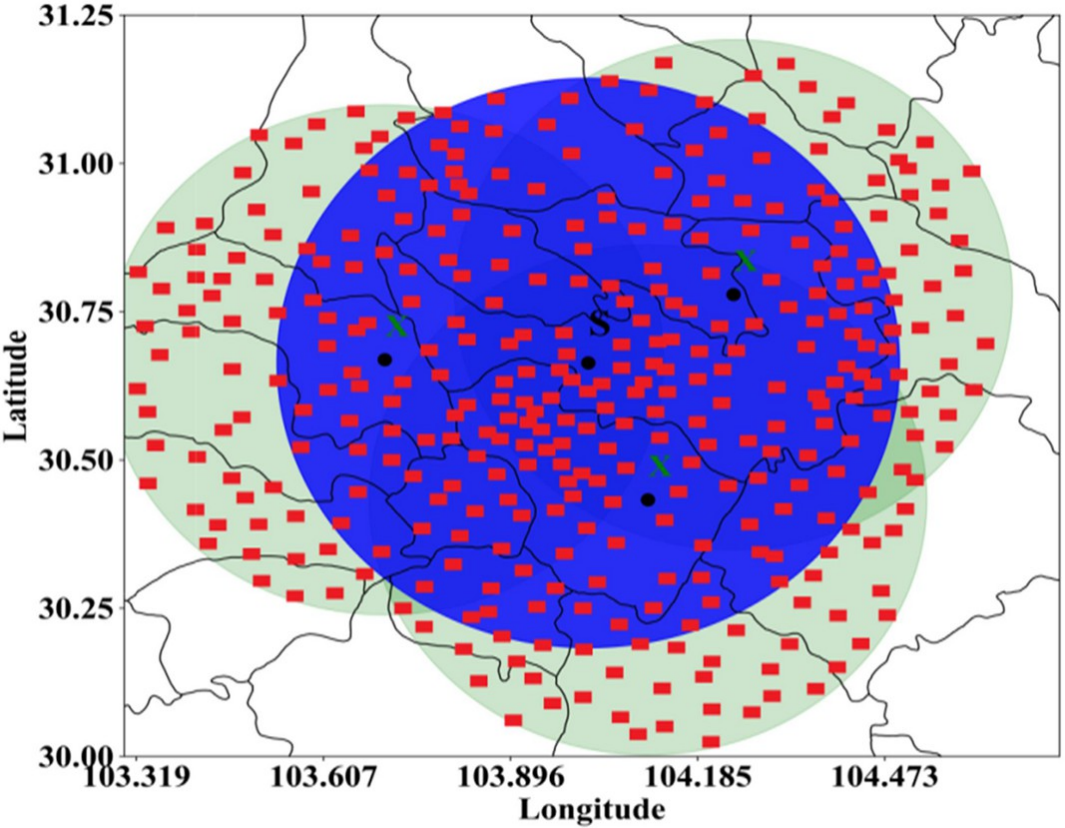
Two types of radars and their corresponding ground automatic station layouts (drawn with Python 3.6).

Fig 10 shows the effective detection coverage of the XPADPRAD network and SDPRDA single radar. The light green area in the image is the range of three XPADPRAD cooperative networking stations, and the blue area is the effective detection range of a single SDPRDA radar. The total detection area of the XPADPRAD network is larger than that of the single SDPRDA radar. On the other hand, the densely distributed red rectangles in the figure are ground-based automatic stations and lightning locators, with a total of 238 groups. They can record whether hail and lightning actually occur on the ground at the same time and in the same location. The introduction of live ground-based automatic station data helps us to conduct a CSI evaluation and RMSDE comparative analysis with the two identification and early warning algorithms.

### Hail and lightning identification warning experiment

**Fig 11:**
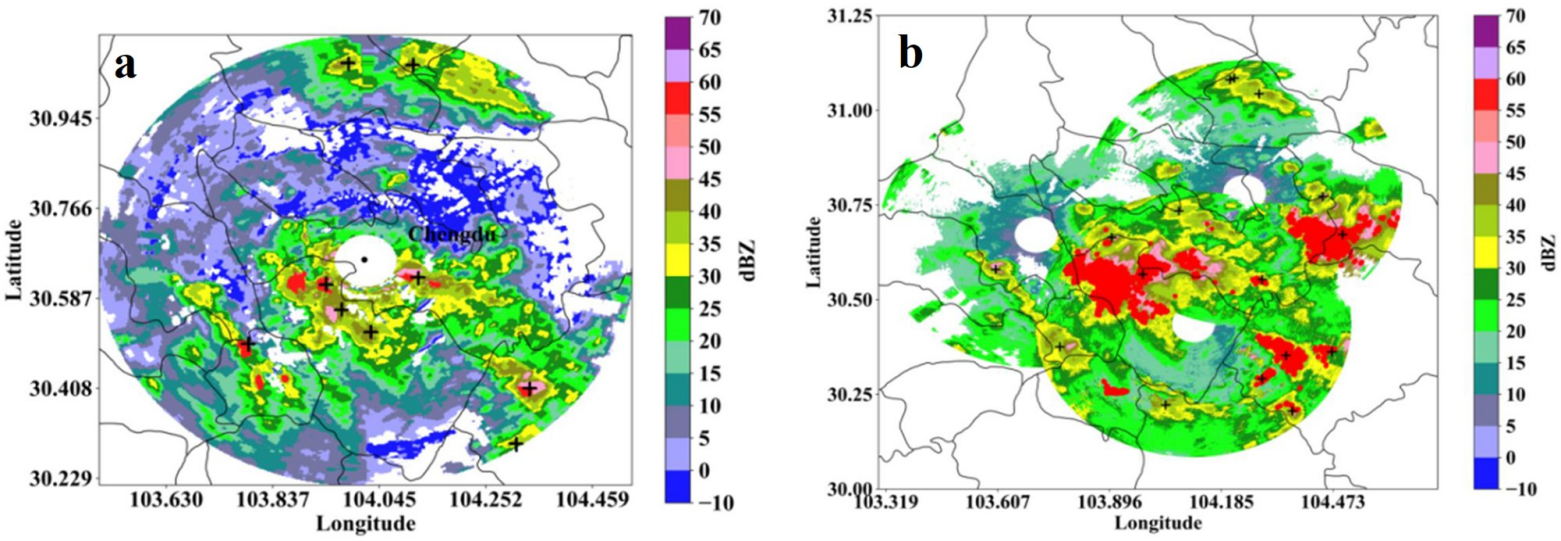
Early warning results of hail and lightning identification of two types of weather radar at 2 km height (drawn with Python 3.6). (a) SRDA single radar. (b) XPADPRAD cooperative networking.

Fig 11 shows the hail and lightning identification and early warning results from the two types of weather radar at a height of 2 km used to monitor extremely strong convective weather events. Fig 11a shows the early warning results of a single SRDA recognition using traditional algorithms. Fig 11b shows the identification and early warning results of the XPADPRAD collaborative networking using the improved algorithm developed in this study. Comparing the two images, it can be clearly seen that the hail recognition range in Fig 11b is significantly larger than that in Fig 11a (red area), which also shows the advantages of multiradar cooperative networking. That is, the filling of the boundary layer and observation blind spots and the data fusion of overlapping areas highlight the consistency of the precipitation spatial structure. From the early warning position of lightning (black ’+’), Fig 11a is basically concentrated in the strong echo position of >35 dBZ, while Fig 11b does not necessarily show a strong echo position for an echo intensity <35 dBZ. The improved method developed in this study adds two important polarization quantities Z_dr_ and ρ_hv_ and introduces the fuzzy logic method for comprehensive probability analysis. Therefore, Fig 11 shows that there is a certain positive correlation between the location of hail and the maximum value of the reflectivity factor, but there is no strong correlation between the probability of lightning occurrence and the maximum value of the reflectivity factor.

The identification and warning outcomes for hail and lightning disastrous weather are objectively assessed with the indices critical success index (CSI), omission alarm rate (OAR) and false alarm rate (FAR) [30], which are defined as follows:

$$\text{FAR}=\frac{\text{false}}{\left(\text{true+false}\right)}$$

$$\text{OAR}=\frac{\text{omission}}{\text{(true}+\text{omission)}}$$

$$\text{CSI}=\frac{\text{true}}{\left(\text{true+false+omission}\right)}$$ (9)

On the right side of the definition of FAR, the numerator is “false”, which means a false alarm grid, and the denominator is “(true+false)”, which means the sum of the success alarm grid and the false alarm grid. On the right side of the equation for OAR, the numerator is “omission”, which presents an omission alarm grid, and the denominator is “(true+omission), which represents the sum of the success alarm grid and the omission alarm grid. On the right side of the CSI equation, the numerator is “true”, which means a success alarm grid, and the denominator is “(true+false+omission)”, which means the sum of the success alarm grid, false alarm grid and omission alarm grid.

**Fig 12:**
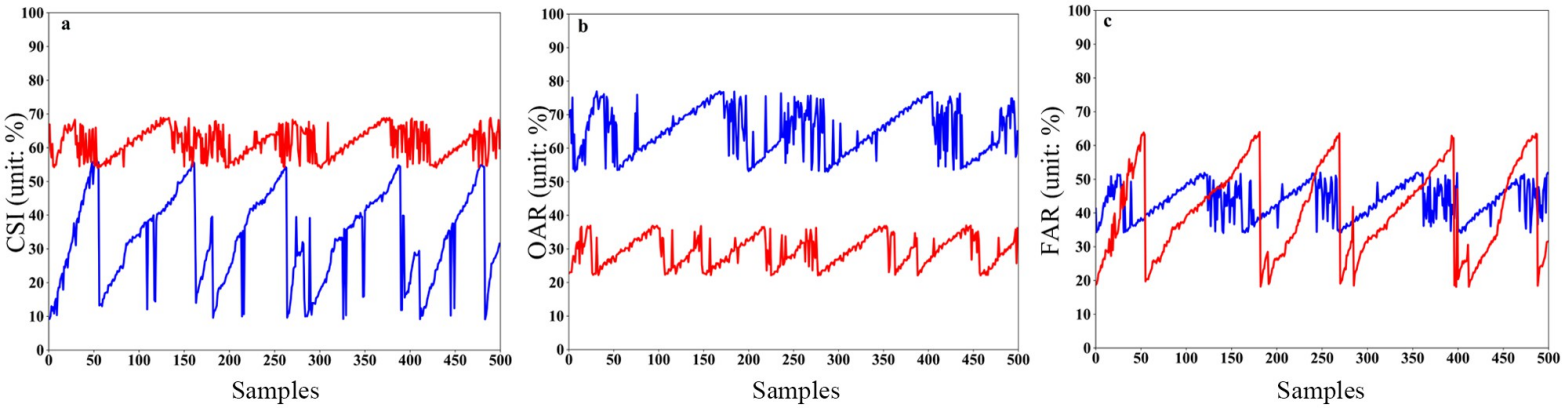
CSI, OAR and FAR scores for hail identification and warning based on two different algorithms. (a) CSI scores, where the blue curve is based on the traditional method and the red curve is based on the modified method. (b) OAR scores, where the blue curve is based on the traditional method and the red curve is based on the modified method. (c) FAR scores, where the blue curve is based on the traditional method and the red curve is based on the modified method.

**Fig 13:**
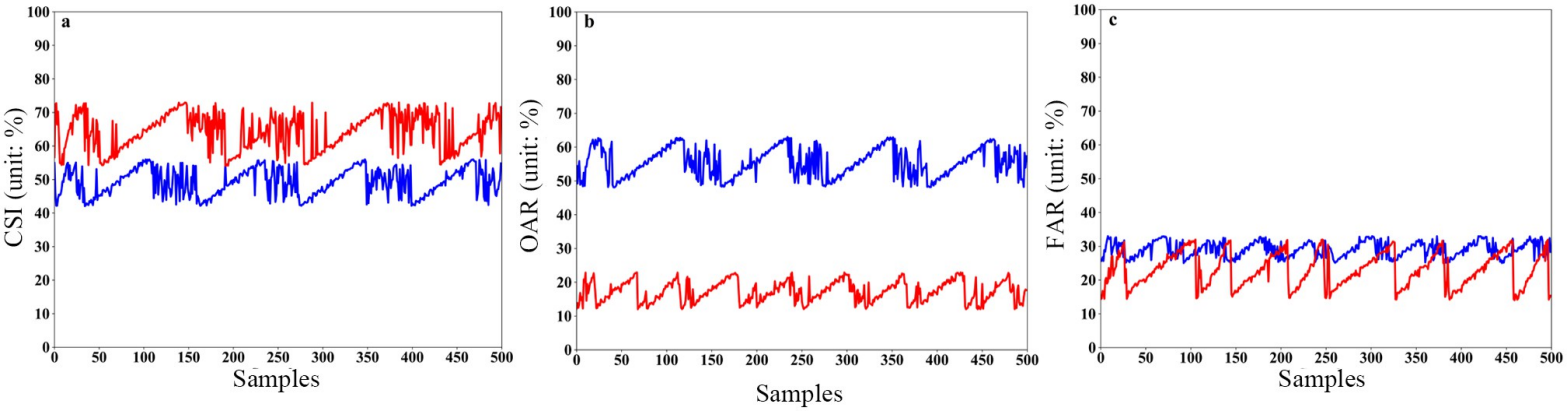
CSI, OAR and FAR scores for lightning identification and warning based on two different algorithms. (a) CSI scores, where the blue curve is based on the traditional method and the red curve is based on the modified method. (b) OAR scores, where the blue curve is based on the traditional method and the red curve is based on the modified method. (c) FAR scores, where the blue curve is based on the traditional method and the red curve is based on the modified method.

A total of 500 sets of radar observations from sample datasets of extreme hail and lightning disaster weather in Chengdu, Sichuan Province, from 2017 to 2022 are collected and then compared with ground-based automatic station and lightning locator live observation data. The CSI/FAR/OAR scores of the two different algorithms for hail and lightning identification and warning results are shown in Figs 12 and 13, respectively.

From the CSI comprehensive score of hail recognition (Fig 12a), the CSI scores of the improved algorithm in this study concentrate between 55% and 70% (red curve), while the CSI scores based on the traditional algorithm (blue curve) show a large range, with the lowest score being <10%. Additionally, the CSI values obtained based on the traditional algorithm are noticeably lower than those obtained from the improved algorithm. Fig 12b shows that the traditional hail recognition algorithm based on SDPRDA has a very high OAR (blue dotted line), which can reach up to nearly 80%, while the improved method based on the XPADPRAD collaborative networking system has a very low OAR (blue solid line), with a minimum of 20%. This further shows that XPADPRAD cooperative networking can effectively increase the observation range, reduce the observation blind area and increase the data continuity. Furthermore, the H_DR_ index is introduced into the improved method, which outperforms the traditional fixed threshold method based on a single Z_h_. From the FAR of the hail recognition results (Fig 12c), the FAR of the traditional algorithm is significantly lower than that of the OAR (blue dotted line) and more concentrated at 30~50%, while the range of the FAR from the improved algorithm is very large (20%-60%; red curve), which indicates that its FAR is very unstable.

In terms of the CSI score, the CSI of the improved algorithm presented in this study is between 54~73% (red curve), while the CSI of the traditional algorithm is between 42~56% (blue curve). Although the CSI scores obtained based on the traditional method are lower than those obtained from the improved method, the gap for lightning is not as large as that for hail. From Fig 13b, we can see that the OAR from the traditional lightning algorithm based on SDPRDA is also high (blue curve), most of which is concentrated between 50% and 60%, while the improved OAR method based on the XPADPRAD collaborative networking system is very low (red curve), and the minimum is close to 10%. This further shows that the algorithm of introducing polarization and generating lightning probability by the fuzzy logic method is much better than the traditional algorithm of judging lightning probability by a single threshold. From the FAR of the lightning recognition results (Fig 13c), the traditional FAR algorithm is generally high (blue curve), and it is most concentrated at approximately 30%, while the FAR from the improved algorithm is relatively low (red curve), with a minimum of 14%.

**Fig 14:**
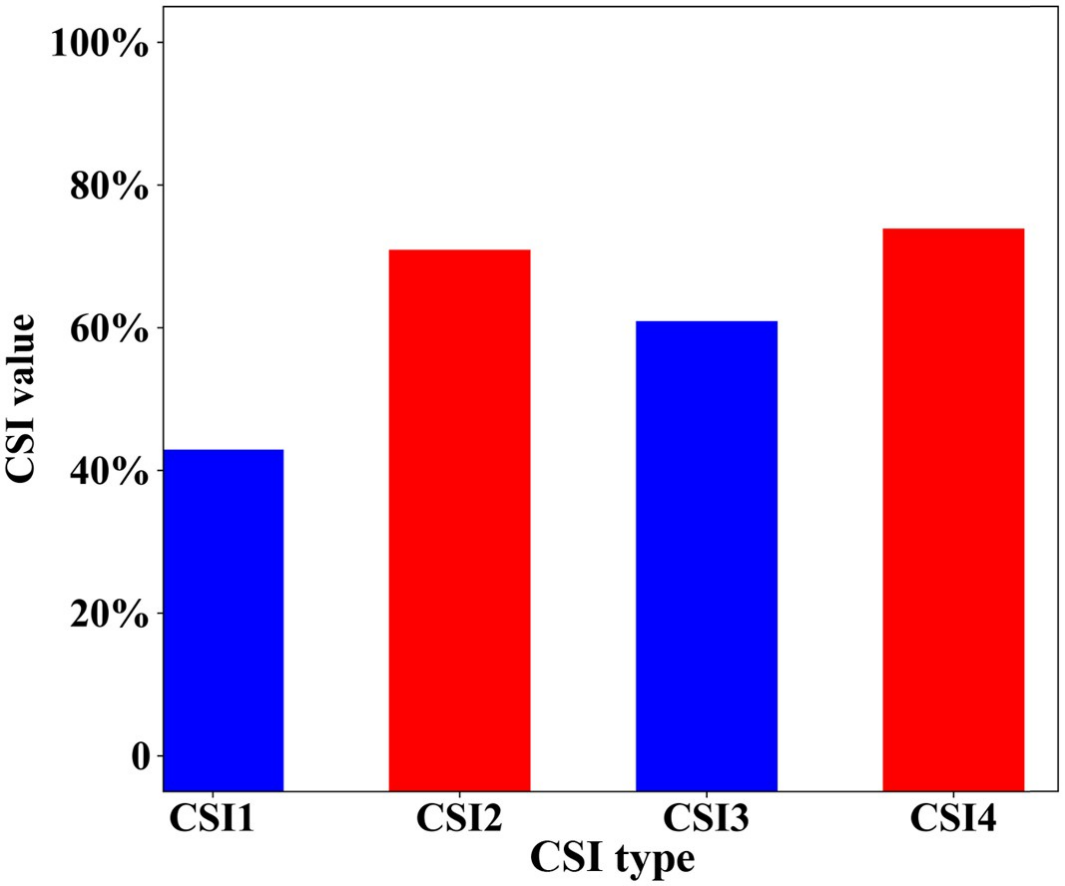
Average CSI, OAR and FAR scores for hail and lightning identification and warning based on 500 samples. (CSI1, average CSI score for hail identification and warning using the traditional method; CSI2, average CSI score for hail identification and warning using the modified method; CSI3, average CSI score for lightning identification and warning using the traditional method; CSI2, average CSI score for lightning identification and warning using the modified method).

Based on the identification results for extreme hail and lightning disaster weather, as shown in Figs 12 and 13, the average CIS scores are obtained (Fig 14). As shown in Fig 14, the average CIS scores of hail and lightning identification and warning obtained from the improved method are better than those based on the traditional method, which are manifested by an increased accuracy in identifying hail (from 33% to 61%) and a higher absolute value of lightning identification precision (up to 64%).

To further compare the accuracy of the two algorithms when identifying hail and lightning locations, we introduce the ARMSE for evaluation. The ARMSE is defined as follows:

$$\begin{array}{l} {\text{ARMSE}}_{\text{hail}}=\frac{\left(\sqrt{\frac{\sum_{i=1}^{N_{\text{1}}}{\left(x_{i}^{h}-\bar{{\text{x}}_{i}^{\text{h}}}\right)}^{2}}{N_{\text{1}}}}+\sqrt{\frac{\sum_{i=1}^{N_{\text{1}}}{\left(y_{i}^{h}-\bar{{\text{y}}_{i}^{\text{h}}}\right)}^{2}}{N_{\text{1}}}}\right)}{\text{2}}\\ {\text{ARMSE}}_{\text{lightning}}=\frac{\left(\sqrt{\frac{\sum_{i=1}^{N_{\text{2}}}{\left(x_{i}^{l}-\bar{{\text{x}}_{i}^{\text{l}}}\right)}^{2}}{N_{\text{2}}}}+\sqrt{\frac{\sum_{i=1}^{N_{\text{2}}}{\left(y_{i}^{l}-\bar{{\text{y}}_{i}^{\text{l}}}\right)}^{2}}{N_{\text{2}}}}\right)}{\text{2}} \end{array}$$ (10)

In Eq 10, the left side of the equation is the ARMSDE of hail and the ARMSDE of lightning. The first term on the right side of the equation is the root mean square error of the hail and lightning positions identified by the algorithm in the zonal direction and the actual position observed by the ground-based automatic station. The second term is the root mean square error of the hail and lightning positions identified by the algorithm in the meridional direction and the actual position observed by the ground-based automatic station. *N*_1_ and *N*_2_ are the actual total number of hail and lightning events observed by the ground-based automatic station at the same time and location as those detected by the radar.

Table 7 shows the ARMSDE of the two methods for hail and lightning location identification for 10 hail and lightning weather cases. The data from the XPADPRDA collaborative network were converted to a grid resolution of 60 m. Table 7 shows that when identifying hail locations, the ARMSDE of the traditional algorithm is larger, and the average deviation from the actual observation position on the ground is approximately 300 m. The ARMSDE of the proposed algorithm is significantly reduced, and the average deviation from the actual observation position on the ground is approximately 100 m. In general, the accuracy of hail locations identified by this algorithm is significantly improved, except for a few cases. For the identification of lightning locations, the ARMSE of the traditional algorithm is very large, and the average deviation from the actual observation position on the ground is more than 1 km. The ARMSE of this algorithm is relatively small, but the average deviation from the actual observation position on the ground is also approximately 750 m. In general, the accuracy of the algorithm developed in this study is better than that of the traditional algorithm, but the accuracy is still much worse for hail. On the one hand, this result may be related to the recognition algorithm, but on the other hand, we need to consider the difference between hail and lightning recognition. The two recognition algorithms are based on the data set with a height of 2 km, which must have errors with the actual ground observation. The hail itself is relatively less affected by the wind field in the process of falling from 2 km to the ground. However, the discharge path of lightning is not perpendicular to the ground, and it follows a very complicated route. Therefore, even if the accuracy of the lightning warning algorithm at a height of 2 km is improved, it may still cause a large error with the actual measurement position on the ground.

**Table 7:** ARMSE of the two methods.

Time (UTC)	Traditional method ARMSDE (m)		The method in this study ARMSDE (m)	
	hail	lightning	hail	lightning
2017-7-10 03:32	322.36	914.21	106.24	805.57
2018-6-04 10:32	428.17	1206.32	172.66	982.91
2018-8-18 12:06	134.76	874.18	170.13	915.36
2019-6-11 04:24	336.92	799.22	214.51	564.62
2019-7-23 06:31	218.73	1115.23	96.36	711.07
2019-7-29 11:45	242.69	1075.76	120.47	842.43
2020-6-16 17:23	147.86	819.33	89.71	514.27
2020-9-12 14:26	357.23	1407.82	125.48	1355.47
2021-7-11 15:17	229.87	1200.07	109.75	1052.35
2022-6-24 19:02	419.96	671.27	147.13	586.71

## Conclusion

Compared with the traditional mechanical VOL mode SDPRDA, the method developed in this study has significant advantages in spatial and temporal resolution and is based on XPADPRAD observation data, and the fast phased array VOL mode can advance the hail and lightning warning time by approximately 7 minutes. XPADPRDA demonstrates advantages in detecting, identifying and warning extreme convective disasters such as tornadoes, hail and strong winds [31, 32]. Based on the literature, this study makes further modifications for its application in fast identification and warning: first, the employment of a three-XPADPRDA cooperative network markedly expands the observation range and thus makes up for the blind spots in single XPADPRDA observation; second, the use of the circular recursive region growing method for TITAN algorithm improvement increases the work efficiency of XPADPRDA; and third, the use of polarization parameters, the hail index and the fuzzy logic method noticeably increases the OAR/FAR/CSI scores and ARMSE accuracy in identifying and predicting hail and lightning.

However, challenges remain for our approach to nearly perfect identification and early warning of hail and lightning weather disasters thus far. As the meteorology assessment demonstrated, the first problem is the quality of the observation data itself. The QC and method of fusing Z_dr_, ρ_hv_ and polarization data from the XPADPRAD cooperative networking radar observation data still need to be improved. The second problem is the lack of historical sample data. Hail and lightning disasters occur less frequently. At present, there have only been dozens of complete observation data sets in the past five years. Therefore, the parameters in the inversion H_DR_ index and membership function are uncertain. The above two problems may lead to underreporting and false reporting. The third problem is that the accuracy of the early warning position must be improved. It is necessary to understand the principle of the process of hail and lightning occurring on the ground at a height of 2 km, such as how the path of falling hail changes under the influence of the wind and the internal physical mechanism of the lightning discharge path. In summary, these problems need to be solved in the future, and we hope this work will serve as a foundation for research in the fields of extreme weather disaster identification and warning.
